# Supplementary material for: Melt-quenched carboxylate metal–organic framework glasses
Source: Nat Commun. 2024 Feb 8;15:1174. doi: 10.1038/s41467-024-45326-8 (PMC10853212; doi:10.1038/s41467-024-45326-8)
Supplement: Supplementary file 1 — Supplementary Information [file 41467_2024_45326_MOESM1_ESM.pdf]

Supplementary Information for

Melt-quenched Carboxylate Metal-Organic Framework Glasses

*Min Hyuk Kim[1], Hwa-Sub Lee[2], Dong-Hyun Seo[3], Sung June Cho[*4], Eun-chae Jeon*[2], and Hoi Ri Moon*[5]*

[1] Department of Chemistry, Ulsan National Institute of Science and Technology (UNIST), Ulsan 44919, Republic of Korea.

[2] School of Materials Science and Engineering, University of Ulsan, 93 Daehak-ro, Nam-gu, Ulsan 44610, Republic of Korea

[3]Major of Nano-Mechatronics, University of Science and Technology, 217, Gajeong-ro, Yuseong-gu, Daejeon 34113, Republic of Korea

[4]Department of Chemical Engineering, Chonnam National University, 77 Yongbong-Ro, Buk-gu, Gwangju 61186, Republic of Korea

[5]Department of Chemistry and Nano Science, Ewha Womans University, Seoul 03760, Republic of Korea

*E-mail: sjcho@chonnam.ac.kr; jeonec@ulsan.ac.kr; hoirimoon@ewha.ac.kr

Supplementary Notes 1. Synthesis of *C*-M-adp

C-Mg-adp was synthesized as previously reported with some modifications.[1] Adipic acid ((HOOC)$(CH_2)_4$(COOH)) (0.053 g, 0.36 mmol) was dissolved in 4 mL of DMA and mixed with a solution of $Mg(NO_3)_2 \cdot 6H_2O$ (0.084 g, 0.33 mmol) in 2 mL of MeOH. The mixed solution was sonicated for 10 min and then heated in a Teflon-lined vessel in an autoclave at 120 °C for 24 h before being cooled to room temperature. Colorless block-shaped crystals formed, which were filtered and briefly washed with the DMA than MeOH. The yield was 48 mg (41%). As-synthesized MOF was then dried overnight in a vacuum oven at 60 °C, resulting in crystals with less solvent but the identical structure, *C*-Mg-adp.

For the synthesis of *C*-Mn-adp, adipic acid (0.054 g, 0.37 mmol) was dissolved in 4 mL of DMA, and mixed with a solution of $Mn(NO_3)_2 \cdot 4H_2O$ (0.072g, 0.29 mmol) in 1 mL of MeOH. The mixed solution was sonicated for 10 min and then heated in an aluminum seal-capped glass jar at 110 °C for 24 h before being cooled to room temperature, resulting in the formation of pale pink crystals.

Similarly, for *C*-Co-adp, adipic acid (0.050 g, 0.34 mmol) was dissolved in 4 mL of *N,N*-dimethylformamide (DMF), and mixed with a solution of $CoCl_2 \cdot 6H_2O$ (0.081g, 0.34 mmol) in 2 mL of EtOH. The mixed solution was sonicated for 10 min and then heated in an aluminum seal-capped glass jar at 100 °C for 24 h before being cooled to room temperature. This resulted in the formation of pink crystals and a purple polycrystalline solid formed.

For *C*-Tb-adp, adipic acid (0.058 g, 0.40 mmol) and $Tb(NO_3)_3 \cdot 5H_2O$ (0.018g, 0.41 mmol) were dissolved in 5 mL of DMF. The solution was sonicated for 10 min and then heated in a Teflon-lined vessel in an autoclave at 100 °C for 24 h before being cooled to room temperature. Colorless crystals formed.

All *C*-M-adp compounds were filtered and briefly washed with their respective mother liquors, and then vacuum dried overnight at 60 °C.

Supplementary Notes 2. Thermal analyses of MOFs

All thermal analyses (TGA, DSC) were performed under inert gas conditions, preventing oxidation during the heating process. The samples were prepared as a dried powder form, but some pore solvents remained in samples.

The TGA was performed at a ramp rate of 10 °C min^{-1} for determine the T_d, using a TGA Q50 from TA Instruments. TG-GC-MS analysis were performed at a ramp rate of 10 °C min^{-1}, using a TG209F1-GCMS from Netzsch.

The DSC were performed using a DSC Q200 from TA Instrument, for evaluated the T_m and T_g of *C*-Mg-adp and *C*-Mn-adp, also, DSC 4000 from PerkinElmer, for calculated the dynamic fragility (m) of *G*-Mg-adp with various heating rate (10-40 °C min^{-1}) and measured the thermal behavior of *C*-M-adp MOFs (10 °C min^{-1}). The T_m and T_g of *C*-Mg-adp and *C*-Mn-adp referred to the onset temperature of the melting peak and the glass transition peak.

Supplementary Notes 3. Characterization of MOFs and MOF glasses

XRPD data were collected on a Bruder D2 phaser diffractometer at 30 kV and 10 mA for Cu Kα (λ = 1.54050 Å), with a step size of 0.02° in 2θ. In-situ variable temperature XRPD (VT-XRPD) were measured on a RIGAKU Smart Lab diffractometer for Cu Kα under N_2 atmosphere.

Solution ^{1}H Fourier-transform (FT) NMR experiments were carried out on a 400 MHz Bruker spectrometer, at the UNIST Central Research Facilities (UCRF) in Ulsan National Institute of Science and Technology (UNIST). All samples were prepared after digestion using deuterium chloride solution (35%, in D_2O) and then dissolved in DMSO-d_6 solvent. Also, Infrared spectra were recorded with a ThermoFisher Scientific iS10 FT-IR spectrometer.

SEM images were obtained on a SU-7000 FE-SEM from Hitachi, at the UNIST UCRF. To decrease charging effects, the samples were sputtered with platinum prior to the measurement.

Total scattering data have been collected on a PANalytical B.V Empyrean X-ray diffractometer at 60 kV and 36 mA (3.6 kW) for Ag Kα (λ = 0.559407 Å, Q max ~ 22 $Å^{-1}$), with a step size of 0.06° in 2θ (scan range: 2-148 °) using GaliPIX3D detector. From these data, pair distribution functions (PDF) in the form G(r) have been calculated by PANalytical HighScore Plus. The PDF data represented the short-range order and long-range order correlation (up to at r ~ 99 Å) for all samples. Published structure for the mother structure of *C*-Mg-adp was used to create the simulated PDF data in PDFgui. A simulated partial PDF was also created in PDFgui, which represents the correlation between specific atoms in the structure.

The gas adsorption−desorption isotherms were measured at 77 K (H_2, N_2) and 273K (CO_2) using liquid nitrogen and ice bath each, on a BELSORP-MAX. Prior to the adsorption measurement, the samples were evacuated at 110 °C under vacuum ($p < 10^{-5}$ mbar) for overnight for *C*-Mg-adp and *G*-Mg-adp.

The XAFS data of Mn-adp were measured with the laboratory X-ray spectrometer QuantumLeap-H2000 from Sigray, operating at 30 kV and 300 W. To determine the atomic structure of Mn-adp, XAFS spectra obtaiend at Mn K edge were analyzed using the Athena and Artemis packages[1,2]. The raw data analysis followed standard procedures using the IFEFFIT software package. The spectra underwent calibration, averaging, pre-edge background subtraction, and post-edge normalization using the Athena program within the IFEFFIT software package. Fourier transformation of the k^3-weighted EXAFS oscillations, $k^3\chi(k)$, from k space to R space, was performed within a range of 3.0–11.0 Å^{-1} to generate a radial distribution function. In the context of XAFS analysis, Wavelet transforms provide an extension to Fourier transforms by effectively separating waveform contributions in both time and frequency domains (or, in the case of XAFS, k and R domains)[3,4]. In the present work, the wavelet-based approach is employed to enhance the qualiative analysis of the coordination environment.

Supplementary Notes 4. Simulation methods for MOFs

In this study, initial calculations on prototypical metal-organic frameworks (MOFs) were performed using Density Functional Theory (DFT) with the Cambridge Sequential Total Energy Package (CASTEP) code (version 22.11).[5,6] The plane-wave ultrasoft pseudopotential method was employed, and the OTF exchange functional was used for the exchange and correlation functional, based on the Generalized Gradient Approximation (GGA) - Perdew-Burke-Ernzerhof scheme.[5] Å cutoff energy of 500.0 eV and a k-point grid of 1 × 1 × 1 were utilized to compute the total energy and bulk modulus of the MOFs.

Force Field (FF) simulations were conducted using the General Utility Lattice Program (GULP), version 6.0. [7,8] Molecular Dynamics (MD) simulations were performed in both NVT (constant number of particles, volume, and temperature) and NPT (constant number of particles, pressure, and temperature) ensembles using modified Nosé-Hoover dynamics[9] as well as the leapfrog Verlet integrator with a time step of 0.5 femtoseconds. The simulations were carried out under various temperature conditions with an external pressure set at 0.1 MPa.

The initial configurations for MD calculations were obtained from fully optimized geometries. Each MD trajectory consisted of a production run lasting 20 picoseconds preceded by an equilibration run of 1 picosecond. Production frames were stored at intervals of every 10 femtoseconds.

Supplementary Figures

a

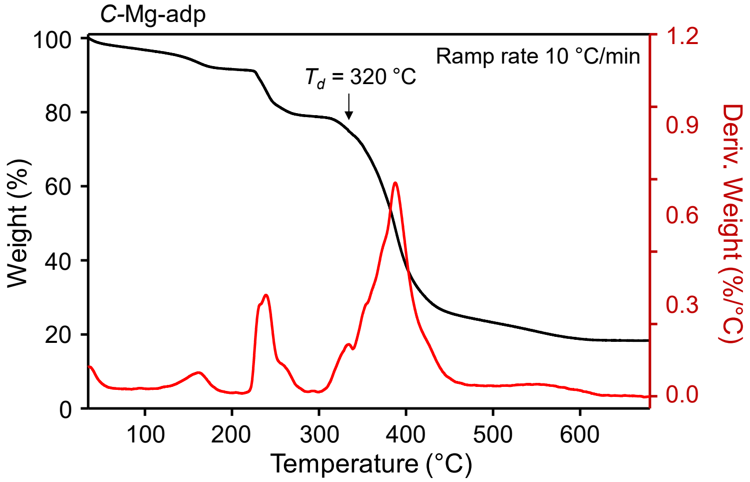

b

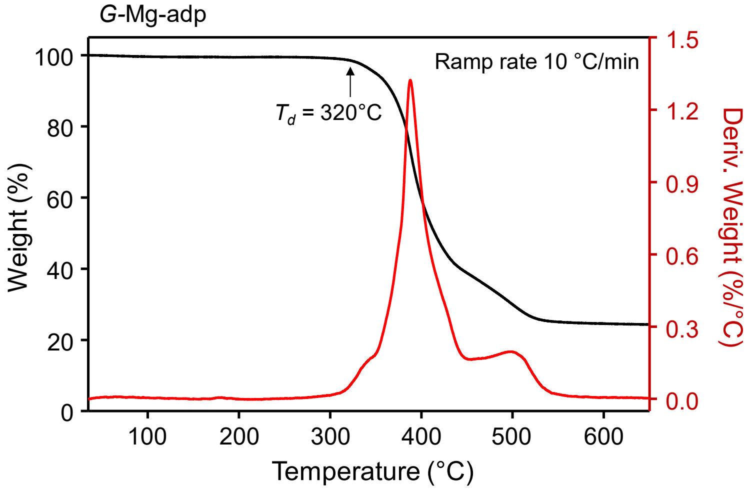

Supplementary Fig. 1 TGA data of **a**, *C*-Mg-adp **b**, *G*-Mg-adp with 10 °C/min ramp rate. T_d was evaluated at 320 °C, which showed abrupt weight loss on TGA.

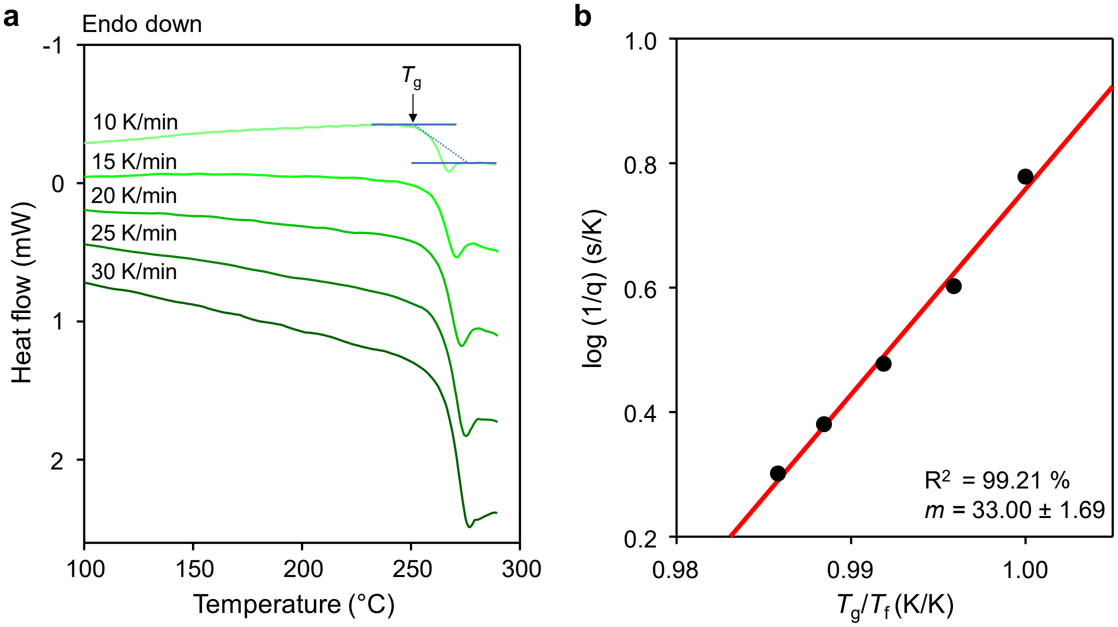

Supplementary Fig. 2 The determination of liquid fragility index (m) from DSC measurements of *G*-Mg-adp. **a**, DSC data using various heating rates. The calorimetric fictive temperature (T_f) obtained using heating rate of 10 K/min is defined as T_g on log (1/q) vs. T_g/ T_f plot. **b**, Fragilities of *G*-Mg-adp, determined as the of log(1/q) vs. T_g/ T_f plot.

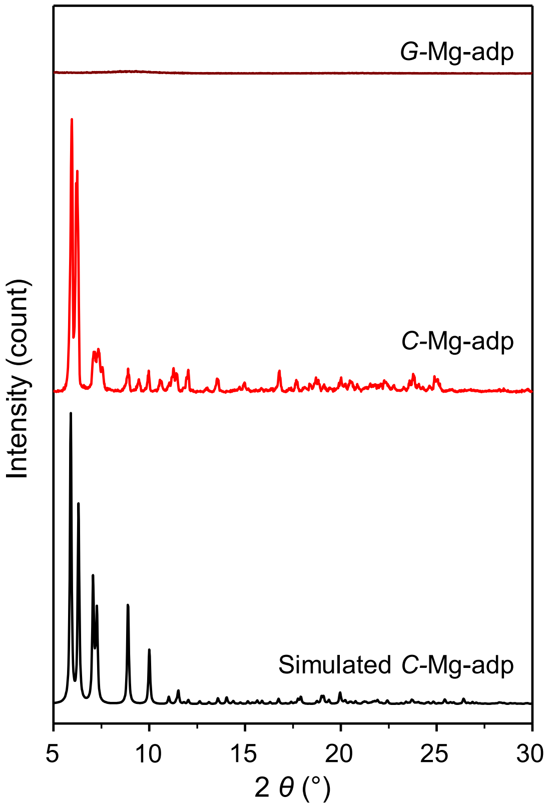

Supplementary Fig. 3 XRPD data of *C*-Mg-adp (red) and *G*-Mg-adp (dark red) with simulated pattern of *C*-Mg-adp (black).

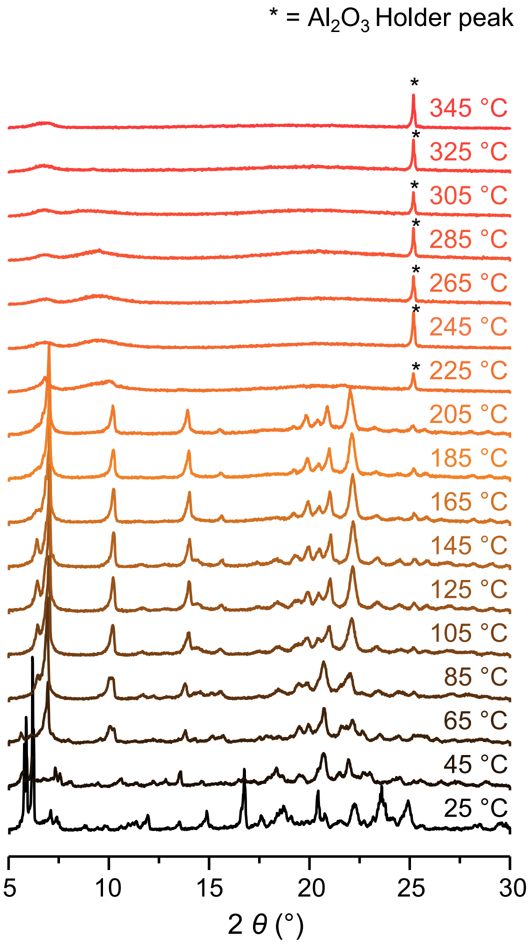

Supplementary Fig. 4 VT-XRPD data of *C*-Mg-adp. The data was measured at 20 °C intervals within the temperature range of 25 – 345 °C in the N_2 inert gas atmosphere.

a

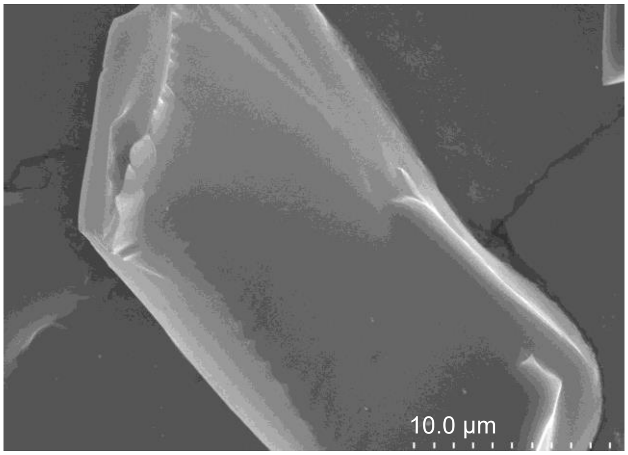

b

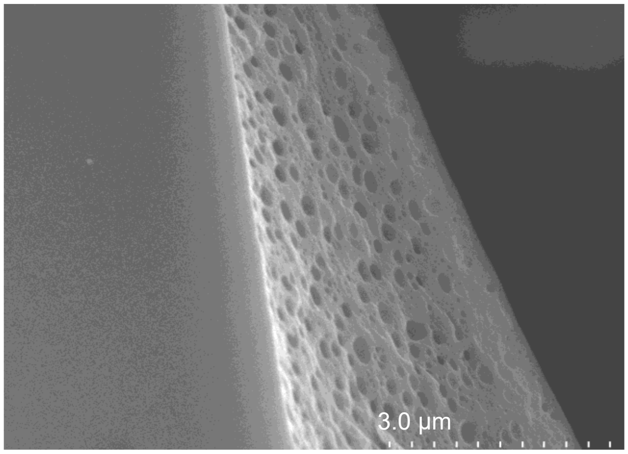

Supplementary Fig. 5 SEM images of *G*-Mg-adp captured after heating *C*-Mg-adp to its melting point (T_m) at 285 °C with a heating rate of 10 °C min^{-1}. **a**, *G*-Mg-adp immediately cooled to room temperature once the temperature reached 285 °C. **b**, *G*-Mg-adp heated at 285 °C for 1 hour and then cooled to room temperature, resulting in the formation of large bubbles due to the evolution of CO gas and organic vesicles.

a

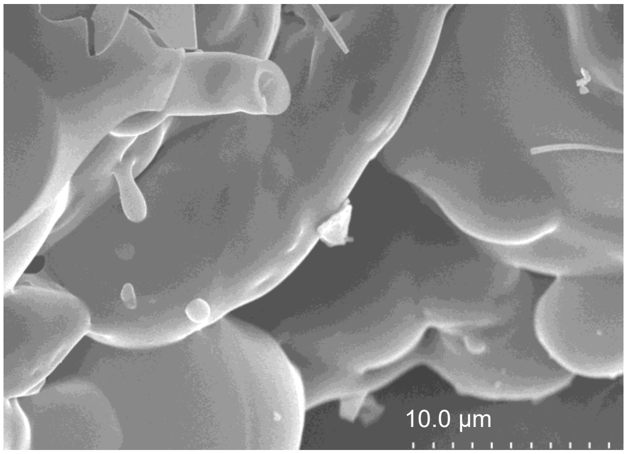

b

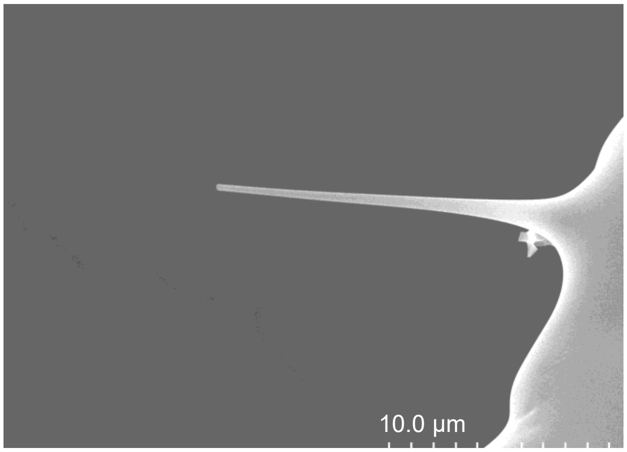

Supplementary Fig. 6 SEM images of *G*-Mg-adp, obtained after quenching and subsequent annealing at 265 °C for 12 hours. **a**, Puffed and **b**, stalactite-like shapes are occurred from viscous fluid, suggesting a melted phase of Mg-adp.

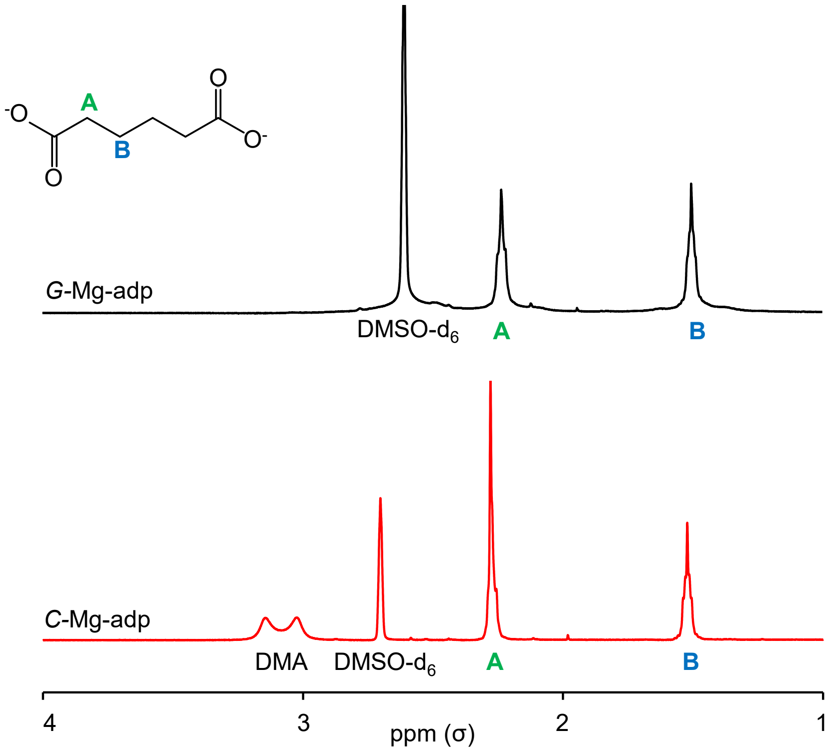

Supplementary Fig. 7 ^{1}H NMR spectra for *C*-Mg-adp (red) and *G*-Mg-adp (black).

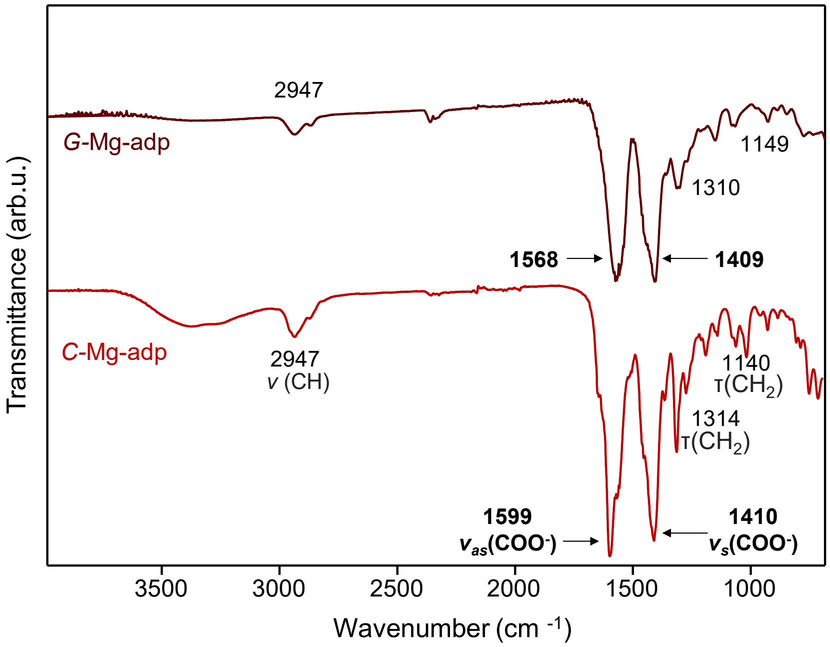

Supplementary Fig. 8 FT-IR spectra of *C*-Mg-adp (red) and *G*-Mg-adp (black). Each term refers to a vibration mode within the adipate ligand.

a

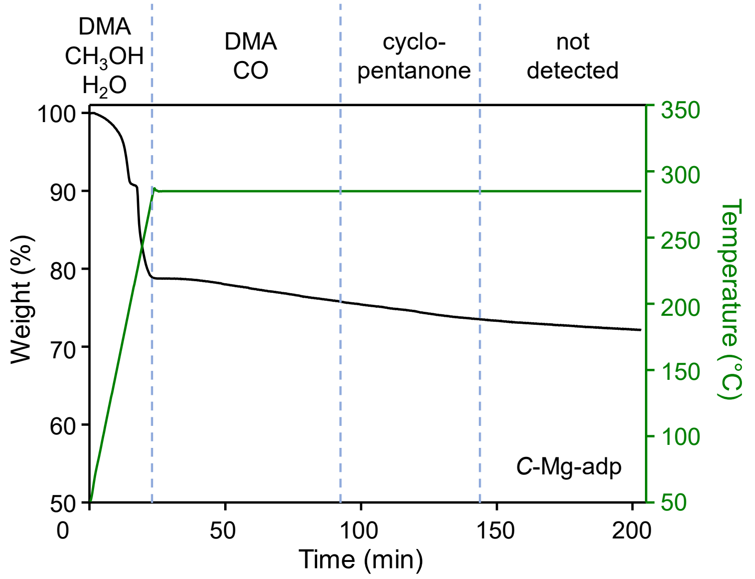

b

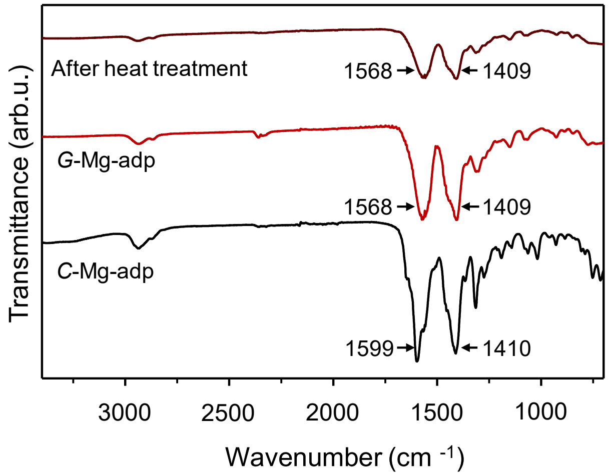

Supplementary Fig. 9 **a**, TG-GC-MS data collected for *C*-Mg-adp by heating up to 285 °C, followed by isothermal measurements for 3 h under an Ar atmosphere. The types of species detected for each range were represented at the top. **b**, The FT-IR data collected after the heat treatment of Mg-adp, compared with *C*-Mg-adp and *G*-Mg-adp.

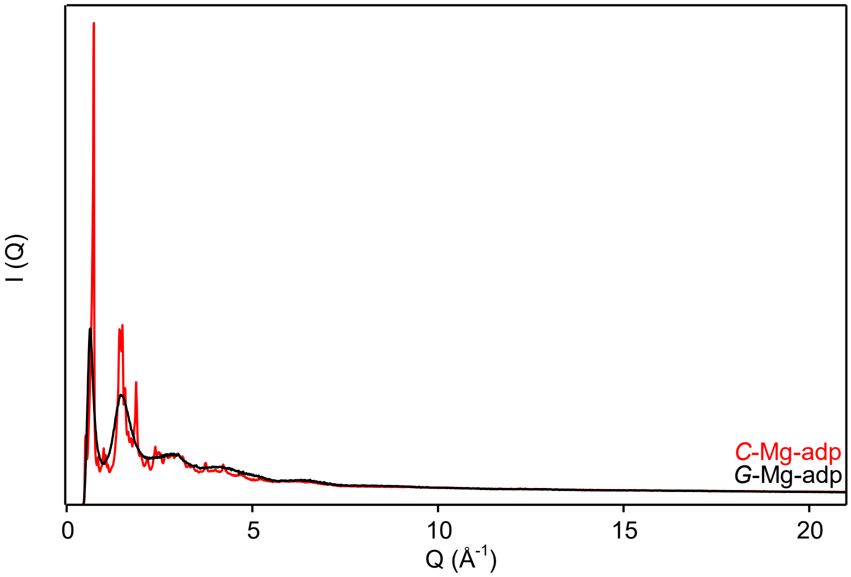

Supplementary Fig. 10 Total scattering data of *C*-Mg-adp (red) and *G*-Mg-adp (black).

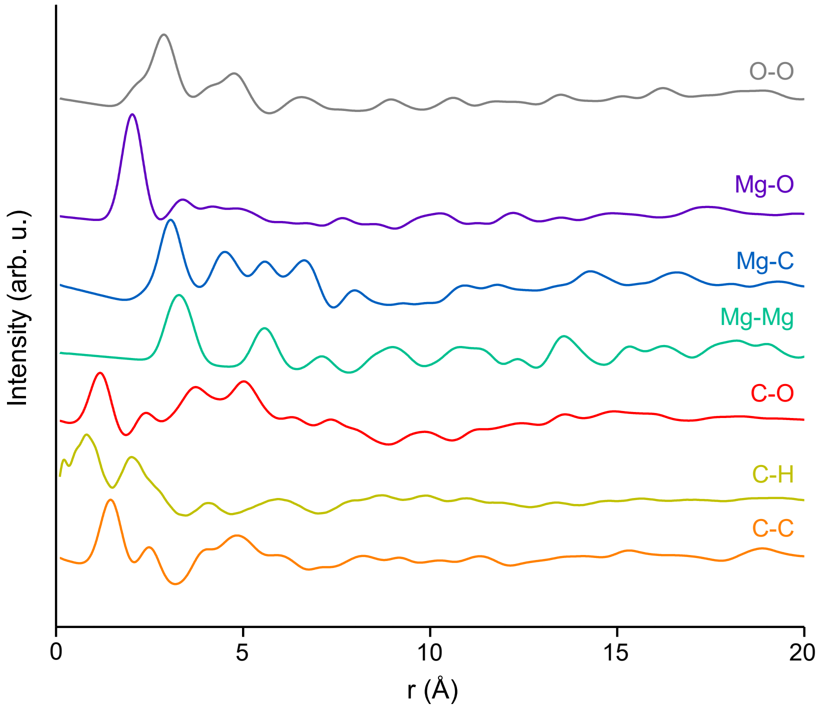

Supplementary Fig. 11 Simulated partial pair distribution functions of *C*-Mg-adp with PDFgui. Each line represents only the correlation between specific atoms.

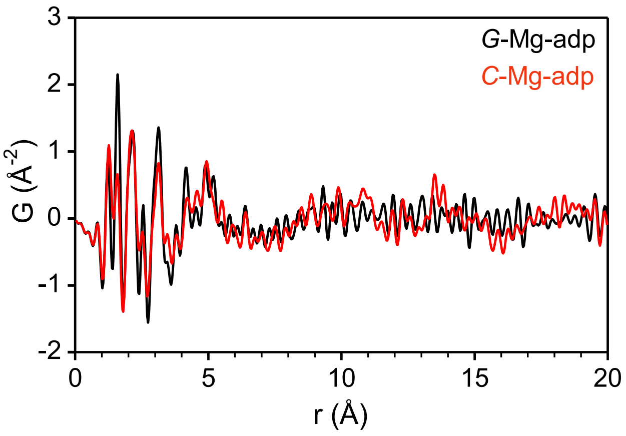

Supplementary Fig. 12 Measured full pair distribution function data for *C*-Mg-adp and *G*-Mg-adp with short-, middle- and long-range correlations shown.

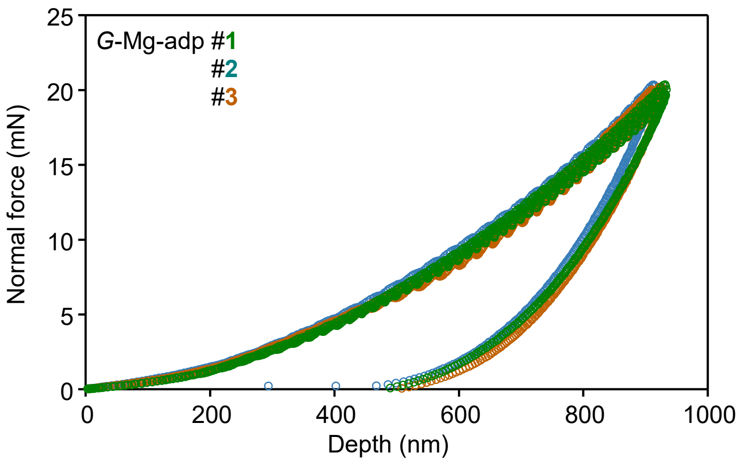

Supplementary Fig. 13 Load-depth curves of *G*-Mg-adp obtained from nanoindentation tests.

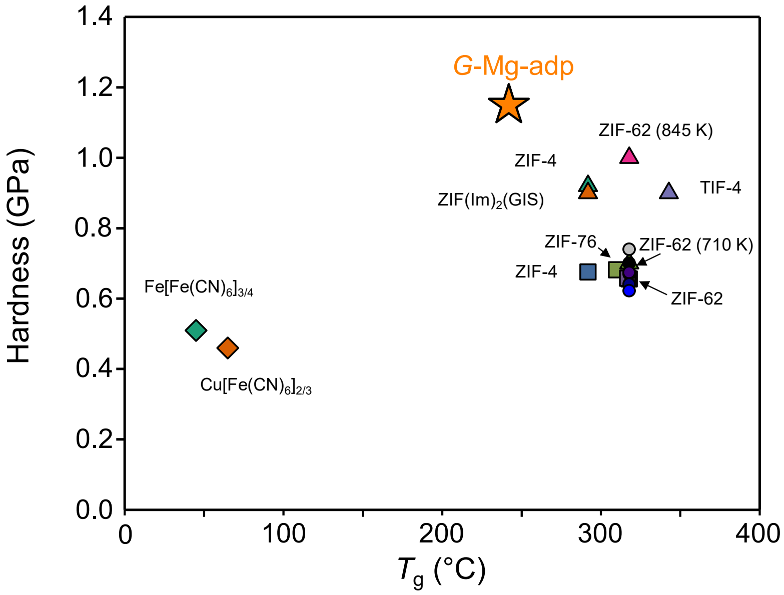

Supplementary Fig. 14 Glass transition temperature-Hardness correlation chart for metal-organic coordination glasses. Note: The circle symbols represent the difference in values according to the change of preparation conditions and hardness test conditions in ZIF-62 glass.

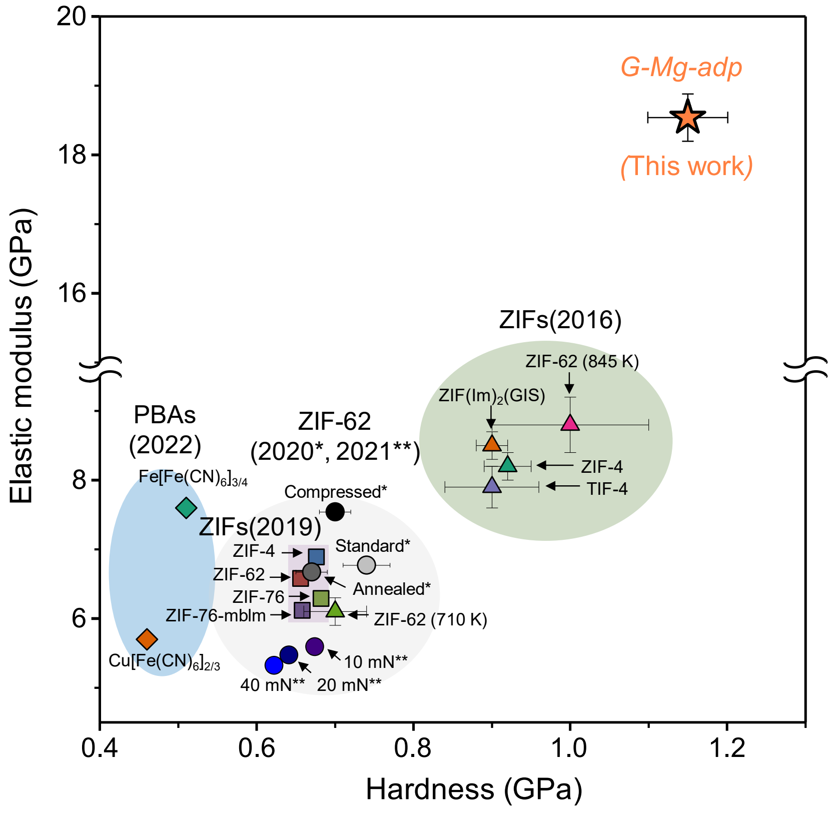

Supplementary Fig. 15 Detail chart of Hardness-Modulus correlation to coordination polymer glasses. Crossed bars indicate the standard deviation for each value. The values in this chart were compiled from the references shown in **Supplementary Table 2**.

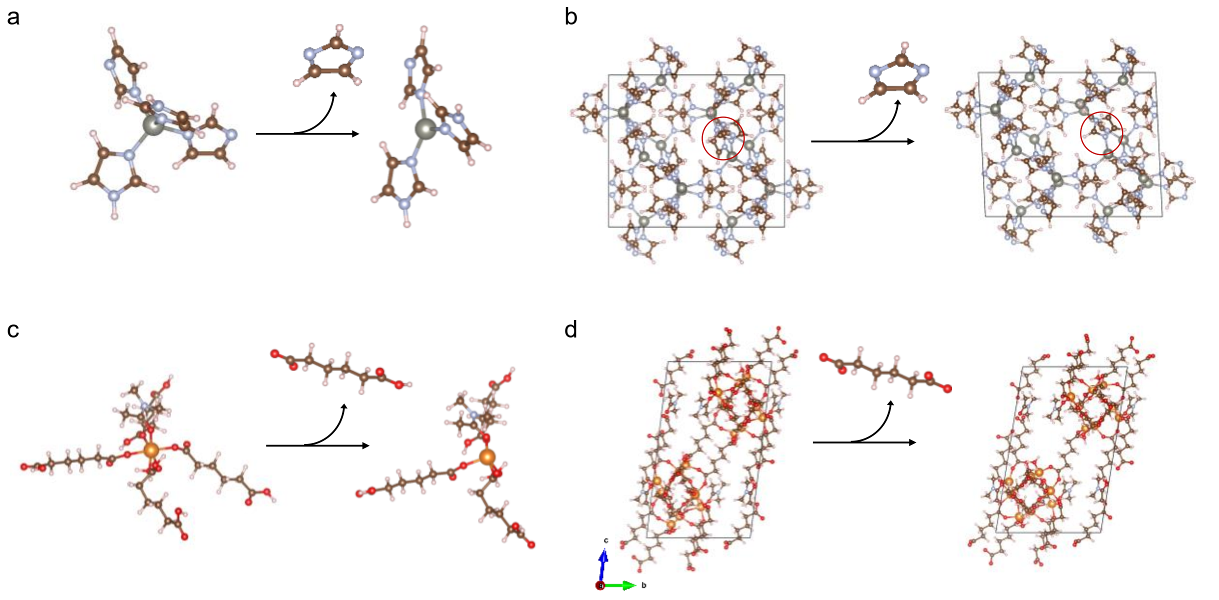

Supplementary Fig. 16 Model construction by removing a ligand from the original structures. **a,** A cluster model and **b,** a crystal model of ZIF-4. **c,** A cluster model and **d,** a crystal model of *C*-Mg-adp.

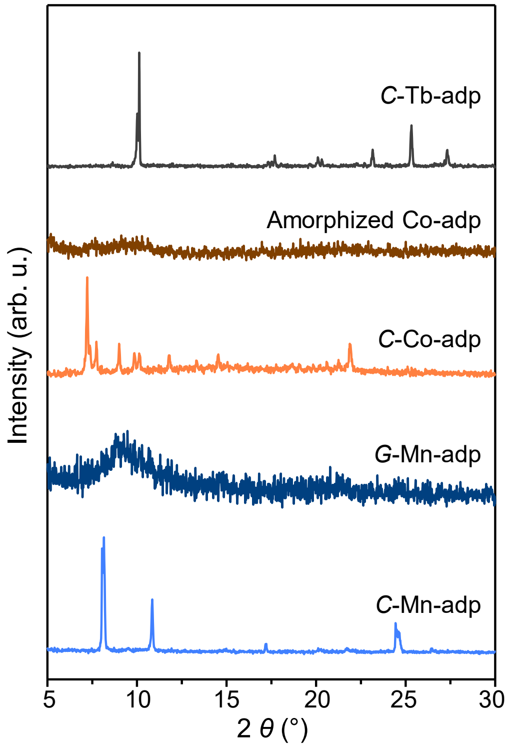

Supplementary Fig. 17 XRPD data of *C*-M-adp (M = Mn^{2+}, Co^{2+}, Tb^{3+}), *G*-Mn-adp and amorphized Co-adp. *G*-Mn-adp was yielded through the melt-quenching process of *C*-Mn-adp under inert gas atmosphere at 240 °C for 10 min. The amorphization of *C*-Co-adp was conducted in the inert gas at 200 °C for 10 min.

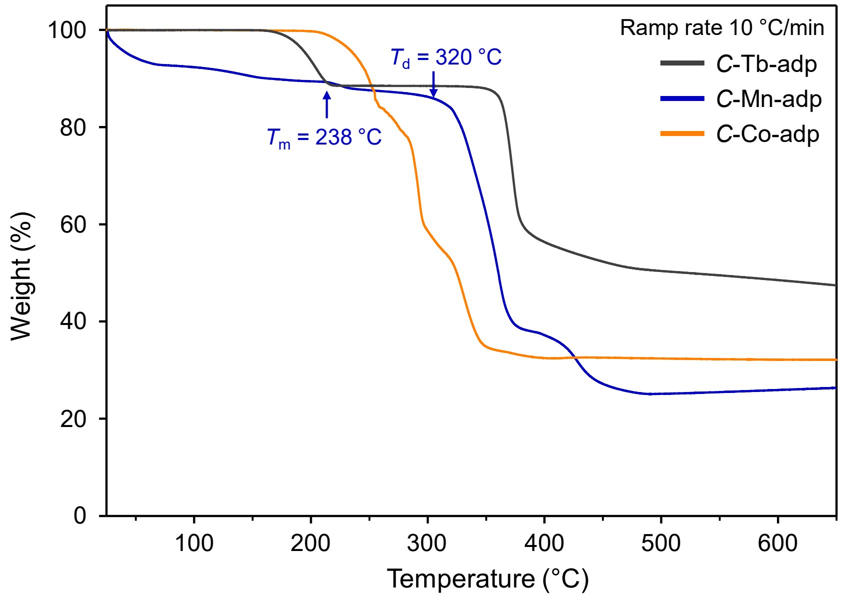

Supplementary Fig. 18 TGA data of *C*-M-adp (M = Mn^{2+}, Co^{2+}, Tb^{3+}). T_m and T_d of *C*-Mn-adp were indicate in the figure.

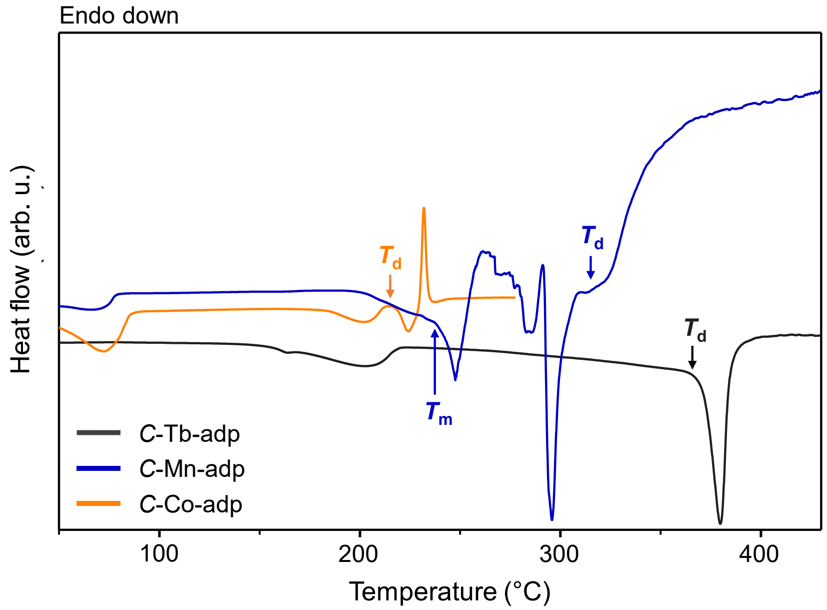

Supplementary Fig. 19 The DSC curves of *C*-M-adp MOFs. *C*-Co-adp (orange), *C*-Tb-adp (black), and *C*-Mn-adp (blue). T_m of *C*-Mn-adp and T_d of each *C*-M-adp were indicated in the figure, respectively.

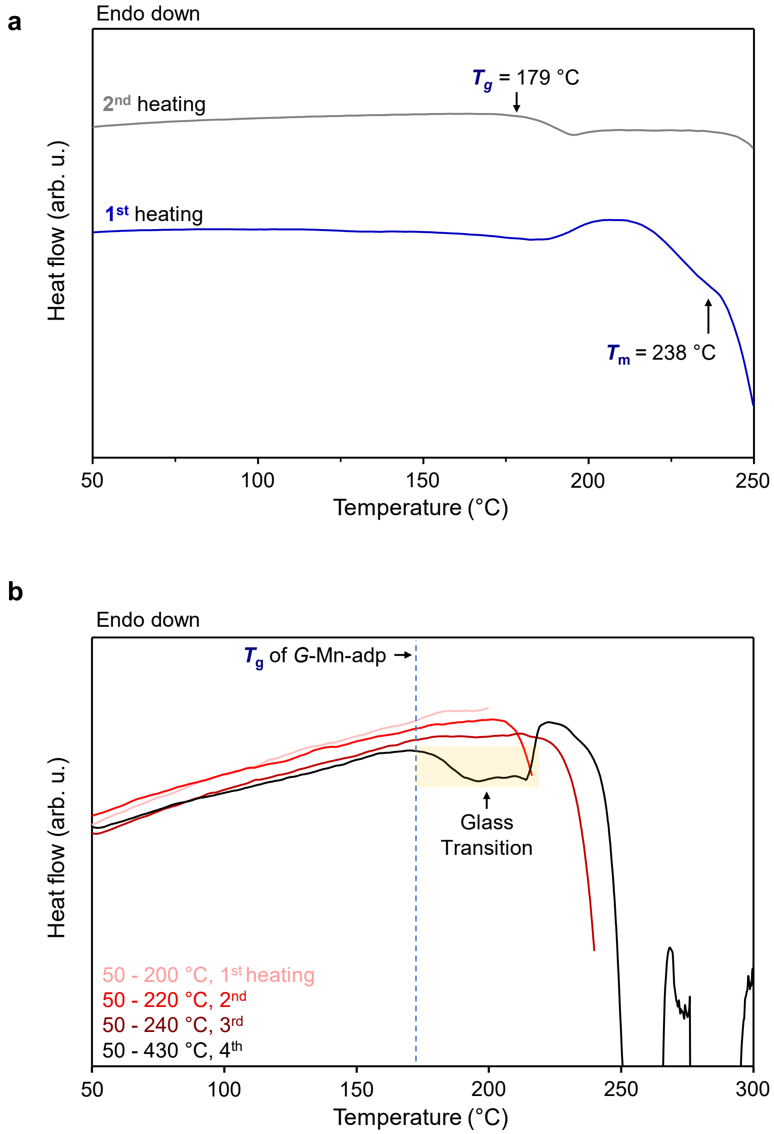

Supplementary Fig. 20 **a**, The DSC curves of *C*-Mn-adp depicts the heating, cooling, and subsequent reheating cycles. Both heating and cooling processes were carried out at a ramping rate of 10 °C/min, with the initial heating phase reaching 250 °C. **b**, Consecutive DSC runs with progressively higher maximum temperatures: 200°C, 220°C, 240°C, and 430°C. Upon reaching each specified maximum temperature, *C*-Mn-adp was maintained under isothermal conditions at that temperature for 5 minutes, followed by a cooling step. A glass transition of Mn-adp can be observed only after reaching at least 240°C, as indicated by the 4th curve at 430°C.

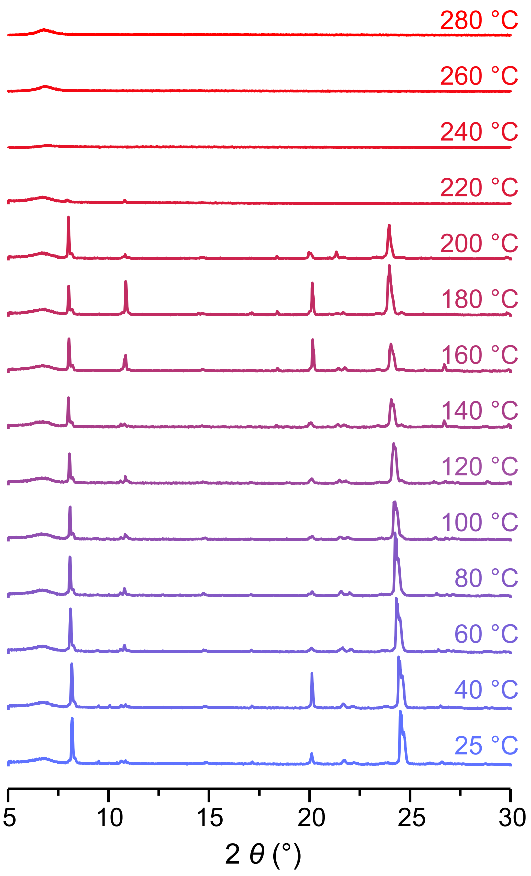

Supplementary Fig. 21 VT-XRPD data of *C*-Mn-adp. The data was measured at 20 °C intervals within the temperature range of 25 – 280 °C in the N_2 inert gas atmosphere.

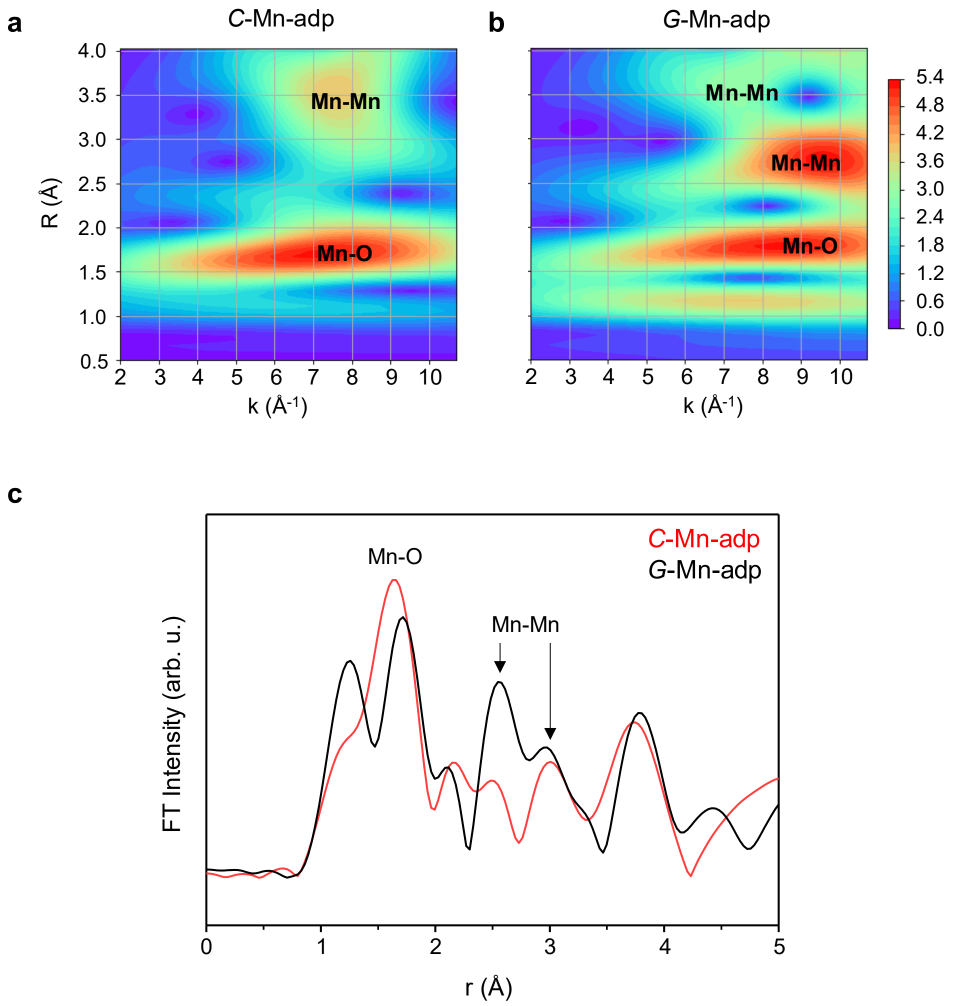

Supplementary Fig. 22 The wavelet contour using Morlet method with η = 10 and σ = 1 over XAFS data of **a**, *C*-Mn-adp and **b**, *G*-Mn-adp. **c**, The Fourie-transforms of XAFS spectra as a function of radial distance for *C*-Mn-adp (red) and *G*-Mn-adp (black). Phase shift within the data set was uncorrected.

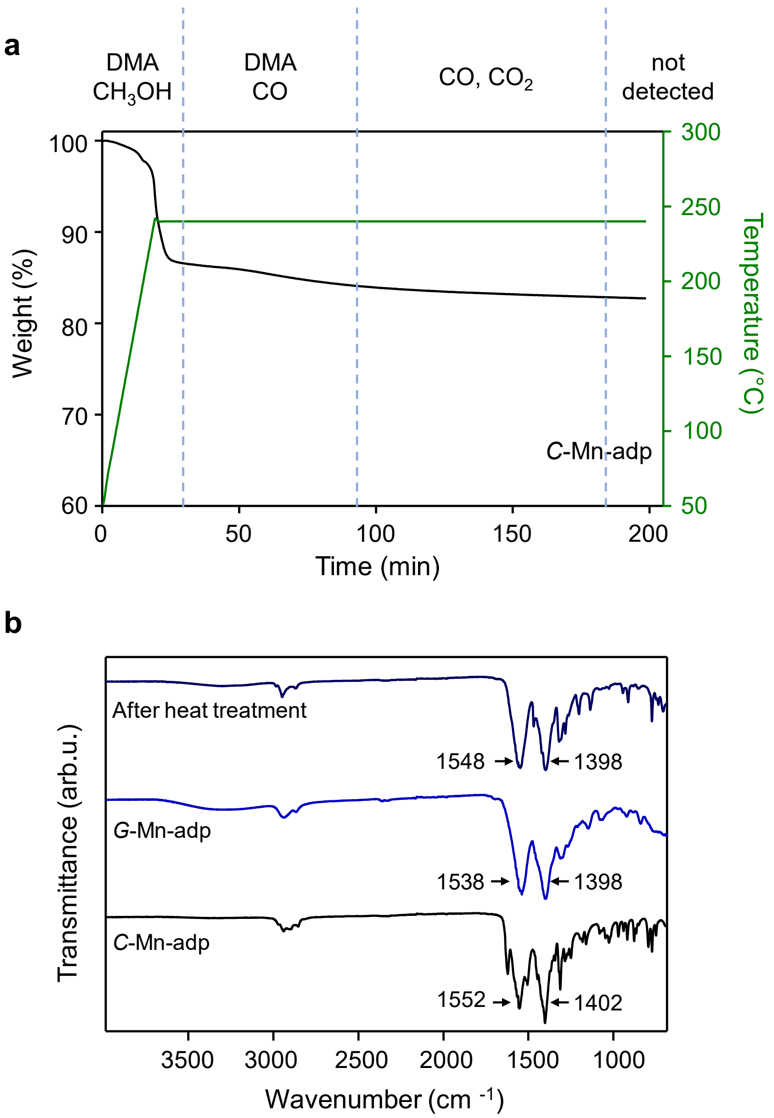

Supplementary Fig. 23 a, TG-GC-MS data were collected for *C*-Mn-adp by heating up to 240 °C, followed by isothermal measurements for 3 h under an Ar atmosphere. The types of species detected for each range were represented at the top. **b**, The FT-IR data collected after the heat treatment, compared with *C*-Mn-adp and *G*-Mn-adp.

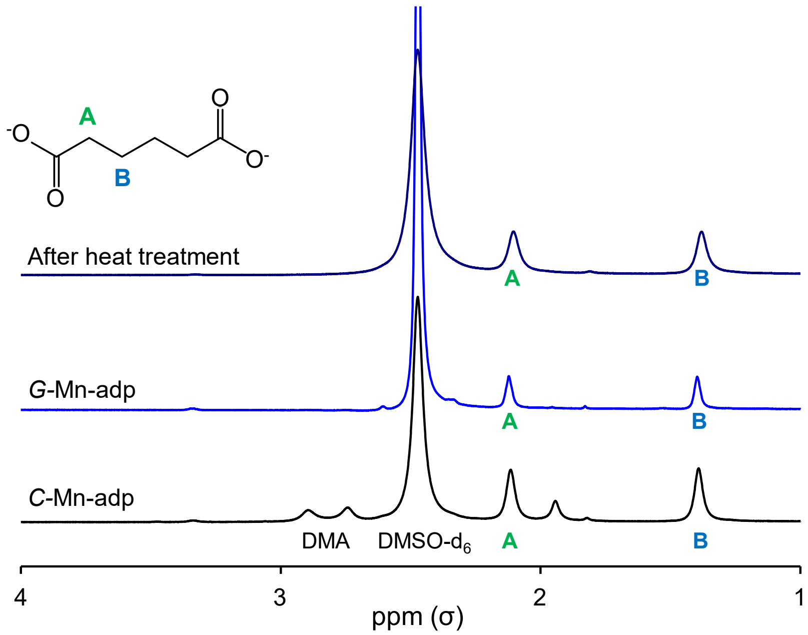

Supplementary Fig. 24 ^{1}H NMR spectra for *C*-Mn-adp, *G*-Mn-adp and the sample after heat treatment at 285 °C for 3 h.

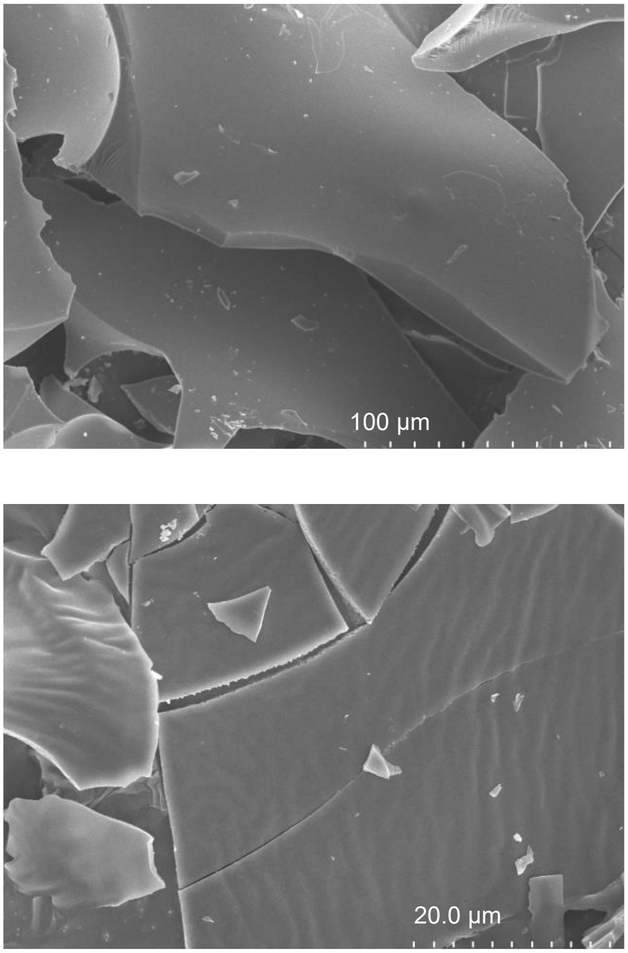

Supplementary Fig. 25 SEM images of *G*-Mn-adp subjected to quenching immediately after reaching its T_m.

a

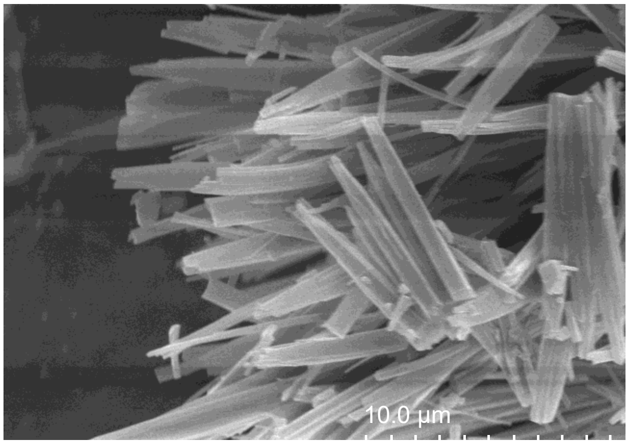

b

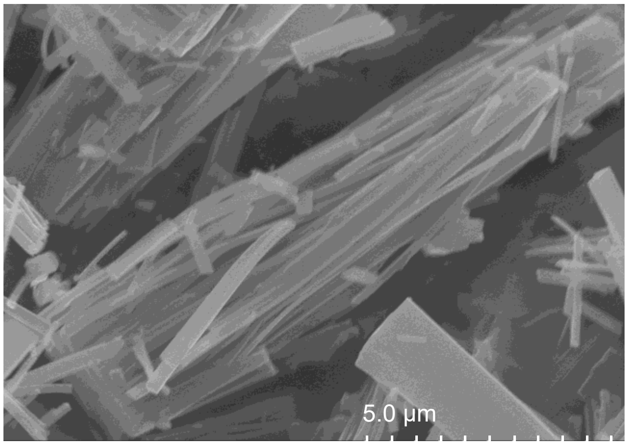

Supplementary Fig. 26 SEM images of **a**, *C*-Co-adp and **b**, thermally amorphized phase, which was heated at 220 ℃ for 10 minutes.

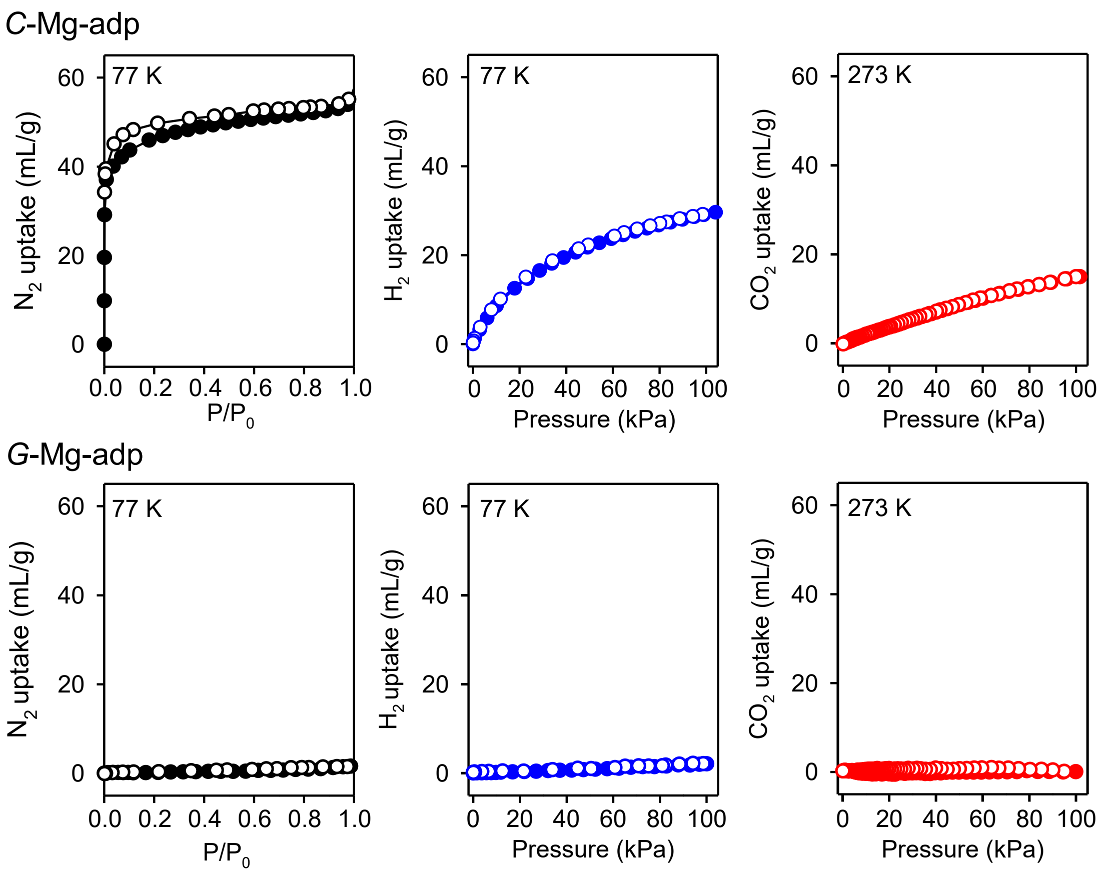

Supplementary Fig. 27 Gas adsorption measurements of *C*-Mg-adp (top) and *G*-Mg-adp (bottom), isotherm data (Inset: isotherm temperature) for N_2 (left), H_2 (middle), and CO_2 (right), with filled and hollow circles represent adsorption and desorption points, respectively.

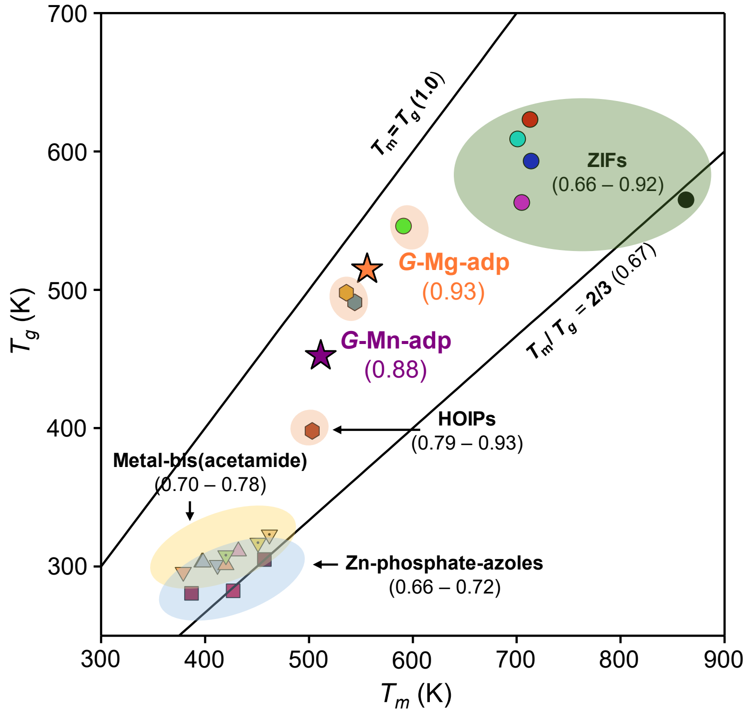

Supplementary Fig. 28 The T_m–T_g correlation chart displays the GFA of meltable coordination structures. Values in parentheses represent the T_m/T_g for each structure. The black lines denote the Kauzmann "2/3" law and the upper bound where T_g equals T_m.

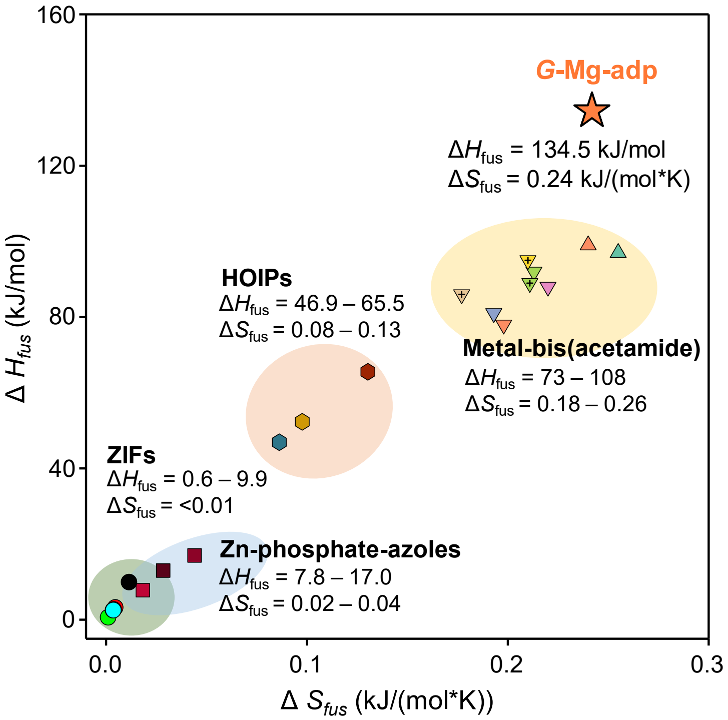

Supplementary Fig. 29 The ΔS_{fus} – ΔH_{fus} correlation for meltable coordination structures.

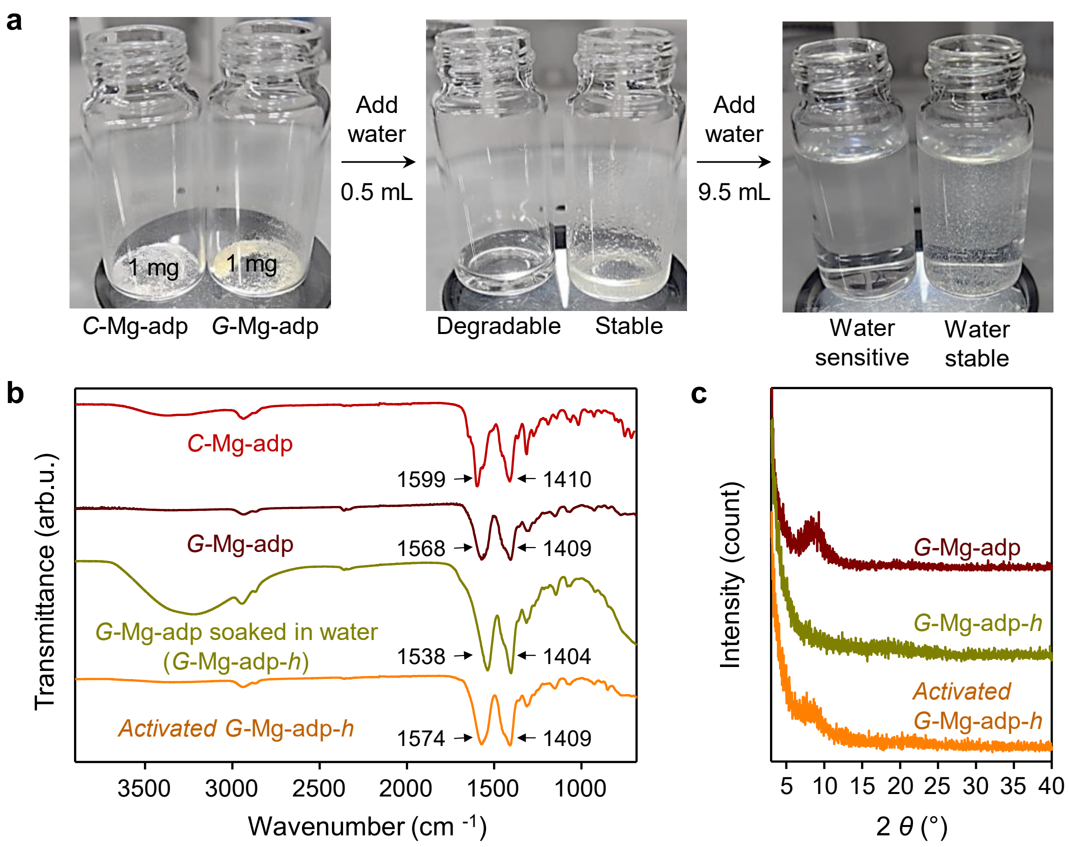

Supplementary Fig. 30 Water stability test of *C*-Mg-adp and *G*-Mg-adp. **a**, Photographs for the water treatment processes for *C*-Mg-adp and *G*-Mg-adp powders. Notably, *G*-Mg-adp remains stable even in a large quantity of water, whereas *C*-Mg-adp undergoes degradation in a small amount of water. **b**, FT-IR data of *C*-Mg-adp, *G*-Mg-adp, *G*-Mg-adp-*h*, and activated *G*-Mg-adp-*h*. **c,** XRPD data of *G*-Mg-adp, *G*-Mg-adp-*h*, and activated *G*-Mg-adp-*h.*

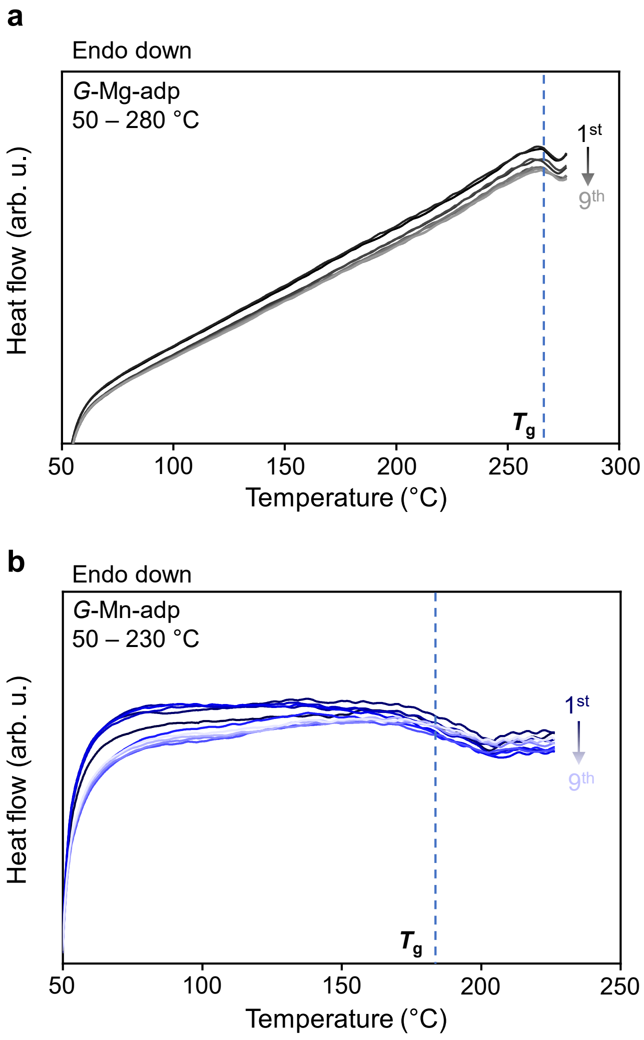

Supplementary Fig. 31 DSC curves of **a,** *G*-Mg-adp and **b,** *G*-Mn-adp from nine consecutive heating and cooling cycles. Only the heating step data is displayed to highlight T_g. Measurements were conducted from 50 °C to slightly below each MOF's T_m, with a heating/cooling rate of 10 °C/min.

Supplementary Tables

Supplementary Table 1. The elastic modulus, hardness, and physical derivatives for various glass materials are presented. The standard deviation is represented within parentheses, and when not mentioned in the reference, it is not indicated. Please refer to the attached references for each symbol to understand the physical meaning of the derivative.

Sample	*H* (GPa)	*E* (GPa)	*H*/*E* (arb. u.)[15]	H^2/E (GPa)[16]	H^3/E^2 (GPa)[17]	$(E^*H)^{1/2}$ (GPa)[18]	Ref.
G-Mg-adp	1.18	18.29	0.065	0.076	0.0044	4.617	This work
ZIF-4	0.92 (± 0.03)	8.2 (± 0.2)	0.112	0.103	0.103	2.746	[19]
ZIF(Im)$_2$ (GIS)	0.9 (± 0.02)	8.5 (± 0.2)	0.106	0.095	0.0953	2.765	[19]
TIF-4	0.9 (± 0.06)	7.9 (± 0.3)	0.114	0.0102	0.103	2.666	[19]
ZIF-62 (710 K)	0.7 (± 0.04)	6.1 (± 0.2)	0.115	0.081	0.0803	2.066	[19]
ZIF-62 (845 K)	1 (± 0.1)	8.8 (± 0.4)	0.114	0.114	0.114	2.966	[19]
ZIF-4	0.676 (± 0.009)	6.89 (± 0.1)	0.0981	0.066	0.0663	2.158	[20]
ZIF-62	0.656 (± 0.005)	6.58 (± 0.02)	0.0997	0.065	0.0654	2.077	[20]
ZIF–76	0.682 (± 0.01)	6.29 (± 0.07)	0.108	0.074	0.0739	2.071	[20]
ZIF-76-mbIm	0.658 (± 0.006)	6.12 (± 0.02)	0.108	0.071	0.0707	2.006	[20]
ZIF-62 (compressed)	0.7 (± 0.02)	7.54 (± 0.03)	0.093	0.065	0.065	2.297	[21]

ZIF-62 (annealed)	0.67 (± 0.02)	6.67 (± 0.02)	0.1	0.067	0.0673	2.114	[21]
ZIF-62 (standard)	0.74 (± 0.03)	6.77 (± 0.02)	0.109	0.081	0.0809	2.238	[21]
ZIF-62 (10 mN)[a]	0.674 (± 0.007)	5.59 (± 0.02)	0.121	0.081	0.0813	1.941	[22]
ZIF-62 (20 mN)[a]	0.641 (± 0.006)	5.47 (± 0.02)	0.117	0.075	0.0751	1.873	[22]
ZIF-62 (40 mN)[a]	0.622 (± 0.005)	5.32 (± 0.02)	0.117	0.073	0.0727	1.819	[22]
$Fe[Fe]_{3/4}$-g[b]	0.51	7.6	0.067	0.034	0.0342	1.969	[23]
$Cu[Fe]_{2/3}$-g[b]	0.46	5.7	0.081	0.037	0.0371	1.619	[23]
Adipic acid (100)	-	11.53(± 0.49)	-	-	-		[24]
Adipic acid (110)	0.3	10.39	0.029	0.0087	0.0087	1.766	[25]

[a] Max load on nanoindentation test

[b] Vickers hardness

Supplementary Table 2. Mechanical properties of *G*-Mg-adp evaluated by nanoindentation tests. Values in parentheses represent standard deviations. Maximum load is 20 mN. Wx values which mean the absorbed energy during deformation were calculated by integrating the load-depth graph in **Supplementary Fig. 12**. W_{total} and $W_{elastic}$ were the area of a loading curve and an unloading curve, respectively. $W_{plastic}$ was a difference of W_{total} and $W_{elastic}$. Numbers in parentheses are standard deviations for three experiments.

W_{total} (J)	64.40 (± 0.1507) $*10^{-10}$
$W_{elastic}$ (J)	28.39 (± 0.9339) $*10^{-10}$
$W_{plastic}$ (J)	36.00 (± 0.8595) $*10^{-10}$
$W_{elastic}/W_{total}$ (arb. u.)	0.441(± 0.014)
Hardness (*H*) (GPa)	1.18 (± 0.051)
Elastic modulus (*E*) (GPa)	18.29 (± 0.342)
H/*E* (arb. u.)	0.065 (± 0.004)
H^2/E (GPa)	0.076 (± 0.008)

Supplementary Table 3. Stabilization energy for metal-ligand formation of ZIF-4 and Mg-adp.

Sample	ΔE (kcal mol^{-1}) [a]	
	Cluster model	Crystal model
ZIF-4	-35.4	-65.3
Mg-adp	-39.0	-113.8

[a] ΔE can be obtained following the equation: $\Delta E = E_{tot} - (E_{MOF^+} + E_{L^-})$

Supplementary Table 4. Lattice parameters for the initial, optimized and defect crystal structure.

		a (Å)	b (Å)	c (Å)	α(∘)	β (∘)	γ (∘)	Bulk Modulus (GPa)
ZIF-4	Initial	15.3073	18.4260	15.3950	90.00	90.00	90.00	-
	Optimized[a]	15.4423	18.4164	15.8302	90.00	90.00	90.00	29.9
	Optimized[b]	13.8823	17.8336	15.3667	90.00	90.00	90.00	0.86/1.44/1.15[c]
	Defect[d]	13.9267	18.5674	15.1760	92.83	89.01	90.37	3.45/1.22/2.33[c]
Mg-adp	Initial	13.4950	19.0163	25.9664	81.04	74.94	69.22	-
	Optimized[a]	13.4990	18.8970	26.0070	80.14	74.93	69.07	13.2
	Optimized[b]	14.2987	16.2385	26.3646	76.61	72.63	64.90	0.28/4.07/2.17[c]
	Defect[d]	14.7082	16.8661	25.5742	74.02	71.49	64.15	-[e]

[a] The structure has been optimized readily using CASTEP program with OTF potential.

[b] The reactive force field has been utilized during the optimization employing *NPT* condition at 300 K with the General Utility Lattice Program.

[c] The bulk modulus (Reuss/Voigt/Hill conventions) contains information regarding the hardness of a material with respect to various types of deformation which are available from the GULP calculation.

[d] The structure has been optimized under the condition that charge in a periodic cell was neutralized with a background and the charge was obtained using iterative algorithms rather than matrix diagonalization.

[e] The defect structure is likely to be fragile because of the defect induced by the deletion of adp ligand and resulted in the negative bulk modulus.

Supplementary Table 5. Metal-ligand ratio of *C*- and *G*-Mg-adp calculated from TGA data. The weight of each sample at 300 °C is set as 100 wt% because all guests have been removed. These datasets in the figure below are identical to those in Supplementary Fig. 1.

	MgO wt% [a]	adp - O wt% [b]	Mg mol%/g [c]	adp mol%/g [d]	Mg:adp Ratio
G-Mg-adp	24.5 %	75.5 %	0.608 %	0.589 %	1:0.969
C-Mg-adp	23.3 %	76.7 %	0.578 %	0.599 %	1:1.036

[a] Remaining weight of the samples heated up to 700 °C

[b] Loss in weight of the samples heated up to 700 °C

[c] Calculated from MgO wt% using the MW of MgO (40.3 g/mol).

[d] Calculated from Adp - O wt% using the MW of adipic acid excluding H_2O (128.1 g/mol).

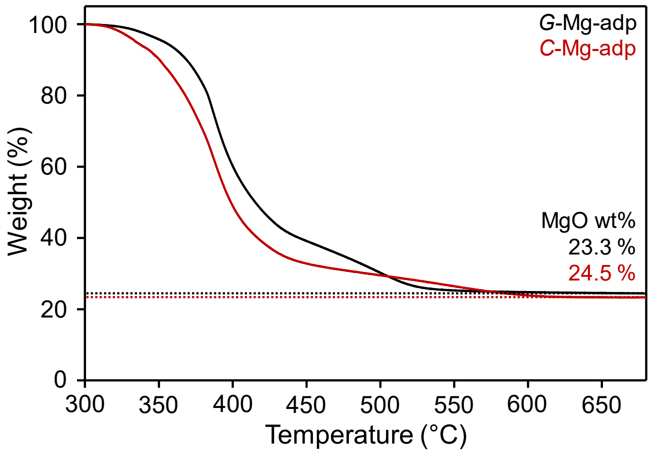

Supplementary References

[1] Ravel, B.; Newville, M. *J. Synchrotron Rad*. 2005, **12**, 537-541.

[2] Newville M. Larch *J. Phys.: Conf. Ser*. 2013, **430**, 012007.

[3] Funke, H.; Scheinost, A. C.; Chukalina, M. *Phys. Rev. B* 2005, **71**, 094110.

[4] Muñoz, M.; Argoul, P.; Farges, F. Continuous Cauchy wavelet transform of XAFS spectra. *Physica Scripta*. IOP Publishing, 2005.

[5] Clark, S. J.; Segall, M. D.; Pickard, C. J.; Hasnip, P. J.; Probert, M. J.; Refson, K.; Payne, M. C. *Z. fur Krist. – Cryst. Mater* 2005, **220**(5-6), 567-570.

[6] Ruiz, V. G.; Liu, W.; Zojer, E.; Scheffler, M.; Tkatchenko, A. *Phys. Rev. Lett*. 2012, 108, 146103.

[7] Gale, J. D. *JCS Faraday Trans*. 1997, **93**, 629.

[8] Cope, E. R.; Dove, M. T. *J. Appl. Cryst.* 2007, **40**, 589-594.

[9] Melchionna, S.; Ciccotti, G.; Holian, B. L. Hoover *Mol. Phys.* 1993, **78**, 533–544.

[10] Farrow, C. L.; Juhas, P.; Liu, J. W.; Bryndin, D.; Božin, E. S.; Bloch, J.; Proffen, T.; Billinge, *J. Phy. Condens. Matter*, 2007, **19**, 335219.

[11] Degen, T.; Sadki, M.; Bron, E.; König, U.; Nénert, G. *Powder Diffr.,* 2014, **29**, S13–S18.

[12] To, T.; Sørensen, S. S.; Stepniewska, M.; Qiao, A.; Jensen, L. R.; Bauchy, M.; Yue, Y.; Smedskjaer, *Nat. Commun*., 2020, **11**, 2593.

[13] Zheng, Q.; Mauro, J. C.; Yue, Y. *J. Non. Cryst. Solids*., 2017, **456**, 95-100.

[14] Qiao, A.; Bennet, T. D.; Tao, H.; Krajnc, A.; Mali, G.; Doherty, C. M.; Thornton, A. W.; Mauro, J. C.; Greaves, G. N.; Yue, Y. *Sci. Adv.,* 2018, **4**, eaao6827.

[15] (a) Finkin, E. Examination of abrasion resistance criteria for some ductile metals. 1974; (b) L.O. Affonso. Machinery Failure Analysis Handbook – Sustain Your Operations and Maximize Uptime. Gulf Publishing Company, USA, 2006.

[16] Oyen, M. L.; Cook, R. F. *J. Mech. Behav. Biomed. Mater*. Elsevier BV, 2009.

[17] Tsui T.Y.; Pharr G.M.; Oliver, W.C.; Bhatia C.S.; White R.L.; Anders A., Brown I.G. *MRS Proceedings*, 1994, **356**, 767.

[18] (a) Karothu, D. P.; Mahmoud Halabi, J.; Ahmed, E.; Ferreira, R.; Spackman, P. R.; Spackman, M. A.; Naumov, P. *Angew. Chem. Int. Ed*, 2022, **134**(10), e202113988.
(b) Labonte, D.; Lenz, A. K.; Oyen, M. L. *Acta biomaterialia*, 2017, **57**, 373-383.

[19] Bennett, T. D.; Yue, Y.; Li, P.; Qiao, A.; Tao, H.; Greaves, N. G.; Richards, T.; Lampronti, G. I.; Redfern, S. A.; Blanc, F. d. r. *J. Am. Chem. Soc*. 2016, **138** (10), 3484-3492.

[20] Li, S.; Limbach, R.; Longley, L.; Shirzadi, A. A.; Walmsley, J. C.; Johnstone, D. N.; … & Bennett, T. D. *J. Am. Chem. Soc* , 2019, **141**(2), 1027-1034.

[21] Qiao, A.; To, T.; Stepniewska, M.; Tao, H.; Calvez, L.; Zhang, X.; … Yue, Y. *Phys. Chem. Chem. Phys*., 2021, **23**, 16923-16931.

[22] Stepniewska, M.; Januchta, K.; Zhou, C.; Qiao, A.; Smedskjaer, M. M.; Yue, Y. *Pros. Natl. Acad. Sci. U.S.A.*, 2020, **117**(19), 10149-10154.

[23] Ma, N.; Ohtani, R.; Le, H. M.; Sørensen, S. S.; Ishikawa, R.; Kawata, S.; ...Horike, S. *Nat. Commun*., 2022, **13**(1), 4023.

[24] Mishra, M. K.; Ramamurty, U.; Desiraju, G. R. *Chem. Asian J*. 2015, **10** (10), 2176-2181.

[25] Mishra, M. K.; Varughese, S.; Ramamurty, U.; Desiraju, G. R. *J. Am. Chem. Soc.* 2013, **135** (22), 8121-8124.

[26] Degen, T.; Sadki, M.; Bron, E.; König, U.; Nénert, G. *Powder Diffr*. 2014, **29** (S2), S13-S18.
